# Guest Editorial: NTP: New Initiatives, New Alignment

**DOI:** 10.1289/ehp.11100

**Published:** 2008-01

**Authors:** John R. Bucher

**Affiliations:** National Toxicology Program, National Institute of Environmental Health Sciences, National Institutes of Health Department of Health and Human Services, Research Triangle Park, North Carolina, E-mail: bucher@niehs.nih.gov

The National Toxicology Program (NTP) has for nearly 30 years led the effort to apply the science of toxicology to the protection of public health. In June 2007 I became the Associate Director of the NTP. I accepted this challenge with enthusiasm and pledged to *a*) maintain our traditional areas of strength in toxicology research and testing; *b*) accelerate our efforts to fulfill the goals set forth in *A National Toxicology Program for the 21st Century: A Roadmap for the Future* (NTP 2004); and *c*) explore new scientific opportunities to understand how individual genetic susceptibilities affect responses to environmental exposures.

The NTP has received considerable attention in the press and from Congress in recent months. Events surrounding the review of bisphenol A by an expert panel convened to provide advice to the NTP Center for the Evaluation of Risks to Human Reproduction (CERHR 2007) led to reviews of all NTP contracts for potential conflicts of interest (NTP Board of Scientific Counselors 2007). The release of the National Institute of Environmental Health Sciences (NIEHS) 2006–2011 Strategic Plan, *New Frontiers in Environmental Health Sciences and Human Health* (NIEHS 2006), also prompted interest in the impacts of changing institutional priorities on the traditional activities of the NTP.

This attention highlighted a long-recognized need for a change in our alignment within the NIEHS Division of Intramural Research (DIR). The need was to give a clearer identity to activities, staff, and dollars associated with the NTP, because for well over a decade these resources had been scattered across several DIR programs. Although the “larger” NTP, as established in 1978, remains an interagency program with our partners at the National Institute for Occupational Safety and Health and at the National Center for Toxicological Research of the Food and Drug Administration, the absence of the NTP from the NIEHS organizational chart had increasingly become a problem for achieving recognition of our primary physical location within the NIH, and also of our unique capabilities and mission.

On 28 October 2007 the DIR was realigned to establish the NTP as a fifth program. The new NTP at the NIEHS brings together the staff working on public health issues and nominated substances, those carrying out the important analysis activities to produce the *Report on Carcinogens* and the NTP–CERHR monographs, and those who support the Interagency Coordinating Committee on the Validation of Alternative Methods.

The budget for this new program will include funding for research and development (R&D) contracts, staff salaries, equipment, and travel. R&D contracts have traditionally provided NTP capabilities for research and testing functions, as well as providing technical and administrative support for analysis activities such as CERHR evaluations. Toxicology efforts carried out by principal investigators in the other four DIR programs will no longer be counted as part of the NTP budget.

The new accounting and staff alignment will provide more transparency to the NTP budget, but it is not a retreat from the successes we have achieved through scientific collaborations between NTP staff scientists and DIR investigators in other programs. In fact, new initiatives will require even more cross-program scientific cooperation and collaboration.

The NTP has studied > 2,500 substances for toxicity and/or carcinogenicity in its nearly 30-year history. Although fewer substances are placed in traditional 2-year rodent cancer bioassays than in prior years, approximately 25–30 new substances are accepted annually for increasingly sophisticated toxicologic evaluations. The budget available for these studies has remained relatively constant; therefore, we are exploring ways in which research and testing programs can be more cost efficient. This is critical if we are to also fund new initiatives.

The realignment provides an organizational scaffold on which to build new or expanded NTP programs in two important areas. The Biomolecular Screening Branch is the new home for activities outlined in the NTP Roadmap (NTP 2004) related to developing new rapid-throughput, robotic assays for screening thousands of chemicals for their capacity to affect a wide variety of biological processes. These assays will probe what were termed “toxicity pathways” in a recent National Research Council report (National Research Council 2007). The program is being developed in close collaboration with the ToxCast Program (U.S. Environmental Protection Agency 2007) and in conjunction with the National Institutes of Health (NIH) Molecular Libraries Initiative NIH Chemical Genomics Center (NIH 2007). In addition, the Biomolecular Screening Branch will house the NTP Screening Core facility, providing somewhat lower-throughput assessments using *Caenorhabditis elegans* and yeast.

The new Host Susceptibility Branch is responsible for the planning and conduct of a program of research aimed at improving our understanding of the genetic bases for differential responses to environmental insults. Building on the recent NIEHS/NTP effort to determine dense single-nucleotide polymorphism maps of 15 commonly used strains of laboratory mice (Frazer et al. 2007), and drawing upon the wide variety of transgenic and knock-out mice now available, staff are devising an innovative approach to collaborative research. This will involve soliciting ideas for specific research projects from academic, government, and possibly private research entities, and using NTP contracts and small grants to perform studies with multiple isogenic lines of genetically defined mice to provide phenotypic data for gene association studies.

I am confident that the research and testing programs of the NTP will continue to provide critical toxicology information on substances of concern to regulatory agencies and the public. Our commitment to public peer review of NTP scientific documents and public input through new nominations and comments to the program is strong. We pledge to continue to work with our agency partners and our scientific advisory boards to provide “good science for good decisions.”

## Figures and Tables

**Figure:**
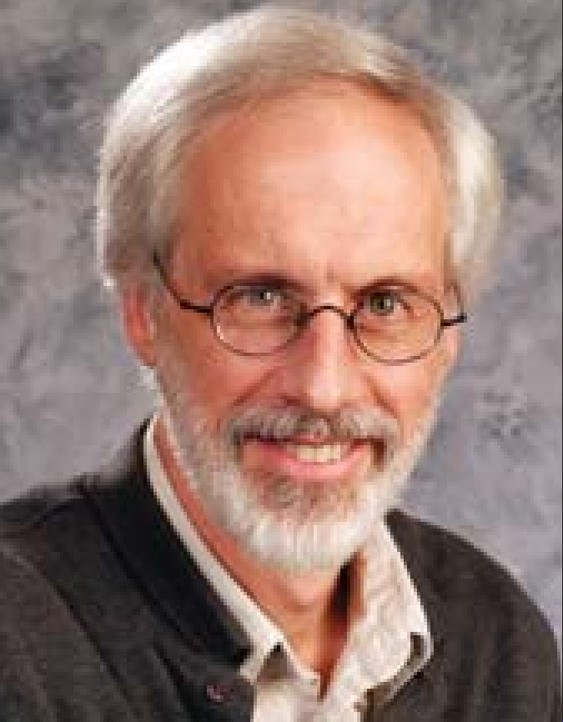
John R. Bucher
